# Protection of Grouper Against *Cryptocaryon irritans* by Immunization With *Tetrahymena thermophila* and Protective Cross-Reactive Antigen Identification

**DOI:** 10.3389/fimmu.2022.891643

**Published:** 2022-07-07

**Authors:** Zequan Mo, Huicheng Wu, Yingtong Hu, Xueli Lai, Wenjie Guo, Yafei Duan, Xueming Dan, Yanwei Li

**Affiliations:** ^1^ University Joint Laboratory of Guangdong Province, Hong Kong and Macao Region on Marine Bioresource Conservation and Exploitation, Guangdong Laboratory for Lingnan Modern Agriculture, College of Marine Sciences, South China Agricultural University, Guangzhou, China; ^2^ Henry Fok School of Biology and Agriculture, Shaoguan University, Shaoguan, China; ^3^ College of Veterinary Medicine, South China Agricultural University, Guangzhou, China

**Keywords:** *Cryptocaryon irritans*, *Tetrahymena thermophila*, tubulin, heterologous vaccine, grouper

## Abstract

Vaccination is an effective method to prevent *Cryptocaryon irritans* infection. Although some vaccines have been developed, large-scale production of these vaccines is costly. Development of a heterogenous vaccine generated by low-cost antigens is an alternative method. In the present study, grouper immunized with *Tetrahymena thermophila*, a free-living ciliate that easily grows in inexpensive culture media at high density, showed protective immunity against *C. irritans* infection. Higher immobilization against *C. irritans* theronts was detected in *T. thermophila*–immunized grouper serum, which suggested the existence of a cross-reactive antibody in the serum. By immunoprecipitation and mass spectrometry analyses, tubulin was identified as a potential cross-reactive antigen between *C. irritans* and *T. thermophila*. Recombinant *T. thermophila* tubulin protein (rTt-tubulin) and its antibody were prepared, and immunofluorescence showed that both *C. irritans* and *T. thermophila* cilia were stained by the anti–rTt-tubulin antibody. Grouper immunized with rTt-tubulin showed a reduced infective rate after the *C. irritans* challenge. An enhanced level of *C. irritans*–binding immunoglobulin M (IgM) antibody was detected in serum from rTt-tubulin–immunized grouper. Moreover, specific antibodies were also found in the mucus and tissue culture medium from rTt-tubulin–immunized grouper. Overall, these findings suggested that vaccination with *T. thermophila* elicits cross-reactive protective immunity in grouper against *C. irritans*, and *T. thermophila* may be a potential heterologous antigen for vaccine development.

## Introduction

*Cryptocaryon irritans* (Brown, 1951), an obligate parasitic ciliate, can infect numerous species of marine fish, indicating a potential threat to marine aquaculture (1). Previous studies have found that sublethal exposure to *C. irritans* elicits immune protection (2, 3). Moreover, injection of inactivated *C. irritans* also provides protective resistance (4, 5). In addition, DNA and recombinant protein vaccines encoded by immobilization antigen (I-antigen) genes have also been developed to control this pathogen (6–8). These findings suggest that vaccination may be an effective method to prevent *C. irritans* infection. Although it is possible to grow *C. irritans* outside the host, the yields are limited (9, 10), and large-scale production of *C. irritans* is difficult (8). In addition, the generation of DNA and recombinant protein–based vaccines is often costly.

Previous research has shown that channel catfish (*Ictalurus punctatus*) immunized with different isolates of *Tetrahymena pyriformis* (Lwoff) cilia provide immune protection against an *Ichthyophthirius multifiliis* challenge, which is a freshwater counterpart of *C. irritans* (11, 12). In addition, Goldfish (*Carassius auratus*) using *I. multifiliis* or *T. pyriformis* for vaccination develop protective immunity against *I. multifiliis* and other parasitic ciliates (13). These findings suggest that cross-reactive protection against parasitic ciliates is induced by these free-living ciliates. Thus, the development of a heterogenous vaccine may be a promising method for controlling diseases caused by these parasitic ciliates.

*Tetrahymena thermophila* is a freshwater free-living ciliate, indicating that the risk of pathogenic contamination can be ruled out. More importantly, *T. thermophila* easily grows in inexpensive culture media at a high density, which is suitable for the large-scale production of vaccines. Our previous study found that a large number of homologous genes (~76%) are shared by *T. thermophila*, *I. multifiliis*, and *C. irritans* (14). Thus, we hypothesized that immunization with *T. thermophila* may provide immune protection for fish against *C. irritans* infection.

In the present study, orange-spotted grouper (*Epinephelus coioides*) immunized with *T. thermophila* showed a lower infective rate when challenged with *C. irritans*, and a higher immobilization was found in immunized fish. Moreover, tubulin was identified as one of the potential cross-reactive antigens between *T. thermophila* and *C. irritans* by immunoprecipitation and mass spectrometry analyses. Immune protection occurred, and a specific antibody against *C. irritans* was present in grouper vaccinated with the recombinant *T. thermophila* tubulin protein. These findings suggested that *T. thermophila* may be an alternative candidate for the development of a heterogenous vaccine against *C. irritans* infection.

## Materials and Methods

### Fish Maintenance

Healthy orange-spotted groupers (28 ± 3.1 g) and golden pompanos (*Trachinotus ovatus*, 235 ± 13.5 g) were obtained from a local farm in Guangdong, China, and they were maintained at 28°C in a flow-through water system (300 L) as previously described (15). Both fish were acclimated for at least 2 weeks and fed with commercial feed twice a day.

### *C. irritans* Propagation and *T. Thermophila* Culture

*C. irritans* used in this study was originally isolated from an infected golden pompano in a local farm of Daya Bay, Guangdong Province, China. The propagation of *C. irritans* was performed as previously described by using golden pompano as the host (16). Briefly, tomonts were collected from the bottom of a harvest unit and incubated in flow-through seawater. Three days after incubation, post-excystment theronts were collected and used to reinfect golden pompanos (10,000 theronts per fish) for 2 h. The golden pompanos were then transferred into the harvest unit for the next round of propagation.

*T. thermophila* strain SCAU07 used in this study was generated in our previous study (8). Briefly, *T. thermophila* strains CU427 (mating type 6) and CU428 (mating type 7) (a gift from Theodore G. Clark, Cornell University) were mated and transformed with pD5H8-GDCI3, which contains a paromomycin-resistant gene. The positive transformed cells were selected by paromomycin. The SCAU07 strain was cultured in NEFF medium (0.25% protease peptone, 0.25% yeast extract, 0.55% dextrose, and 33 µM FeCl_3_) at 30°C for 24 h with shaking (80 rpm) (8).

### Grouper Vaccination and Parasite Challenge

Both *C. irritans* theronts and *T. thermophila* were collected by centrifugation and diluted to 200,000 ciliates/ml with phosphate buffered saline (PBS). Both ciliates were individually emulsified with Freund’s Complete Adjuvant (FCA, for primary vaccination) or Freund’s Incomplete Adjuvant (FIA, for boost vaccination) at a final concentration of 100,000 ciliates/ml. Because *C. irritans* is an ectoparasite and its infection can induce mucosal immune response, herein for vaccination, groupers were subcutaneously injected (IC) with FCA-emulsified *C. irritans* theronts or *T. thermophila* at a dose of 10,000 ciliates per fish. Two weeks after the primary inoculation, groupers were boosted *via* IC injection of FIA-emulsified *C. irritans* theronts or *T. thermophila* at a dose of 10,000 ciliates per fish. Moreover, the control groupers were injected with FCA- or FIA-emulsified PBS.

For the *C. irritans* challenge, 4 weeks after the boost vaccination, groupers were randomly selected from each group and placed into a separate tank with 100 L of seawater. Post-excystment theronts collected within 2 h were added into each tank at a dose of 4,000 theronts per fish. Two hours after the infection, each grouper was transferred into a 15-L clean tank. The numbers of tomonts were recorded from each tank, and the received tomonts relative to the infective dose (4,000 theronts) were considered as the infective rate at day 5 after challenge. Serum samples for the following tests were collected at day 3 after challenge.

### Immobilization Assay

The immobilization assay was perfromed as presiously described (8). Briefly, 50 μl of grouper serum was serially diluted (two-fold dilutions) with seawater and placed into a 96-well plate. For *C. irritans* immobilization, 50 μl of seawater (containing 500 theronts) was added into each well and incubated for 30 min at room temperature. The last well, in which 50% of theronts was immobilized, was considered as the endpoint titer.

### Detection, Immunoprecipitation, and Mass Spectrometry of Cross-Reactive Antigens

**Figure 1:**
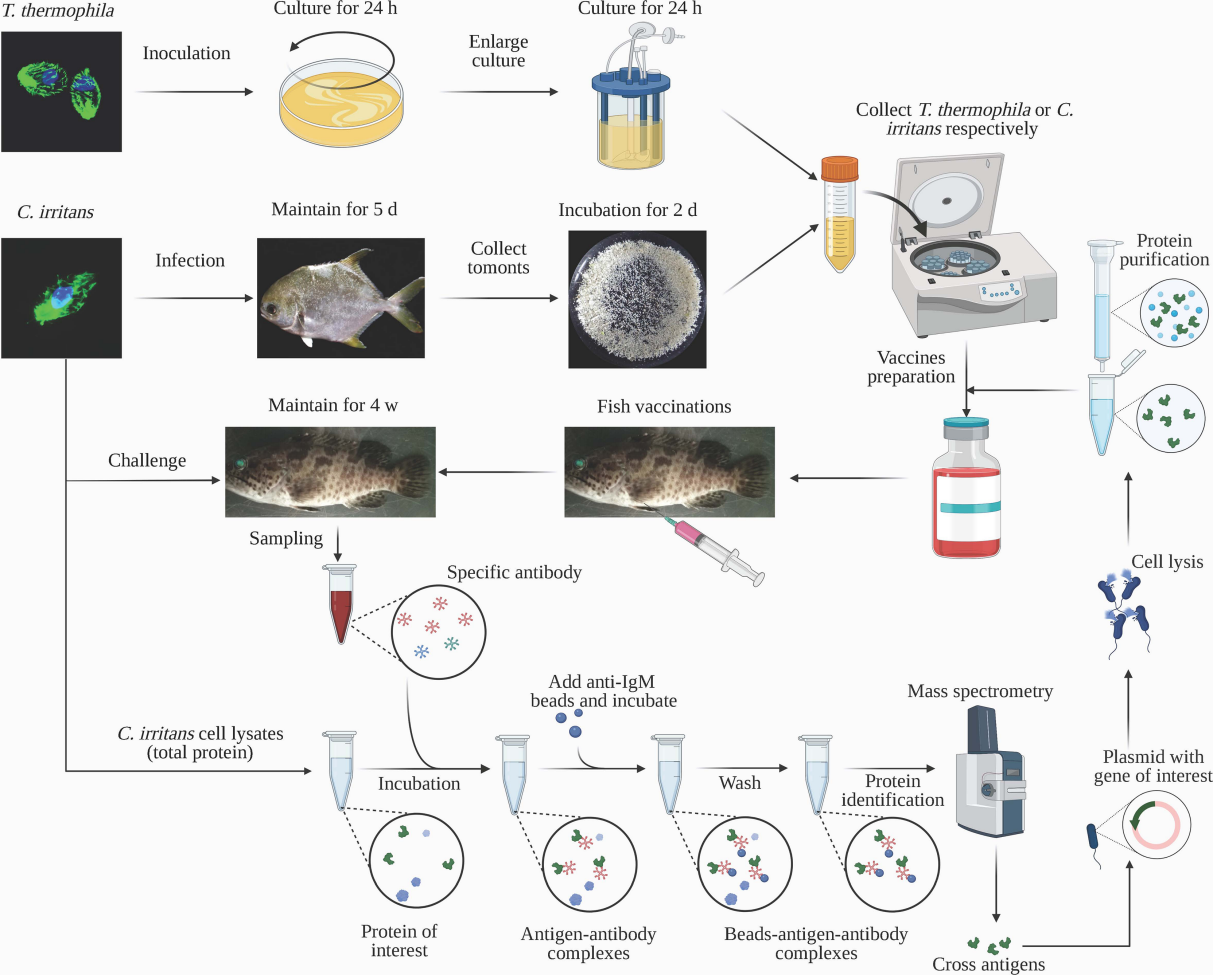
Schematic diagram of heterogenous vaccine development in this study. *C. irritans* and *T. thermophila* were cultured and collected for antigen preparation. After emulsifying with adjuvants, vaccines were individually SQ injected into groupers. Four weeks after the boost vaccination, groupers were challenged with *C. irritans* theronts to measure the infective rate. Serum samples were collected from groupers before the challenge and used for identification of potential cross-reactive antigens by immunoprecipitation and mass spectrometry analyses. The recombinant potential cross-reactive antigen was prepared by prokaryotic expression and injected into grouper for infective rate detection. The schematic was created using BioRender.

Protein samples were isolated from *C. irritans* theronts or *T. thermophila* using RIPA lysis buffer, and the cross-reactive antigens were detected by Western blot analysis. *C. irritans* theront protein samples (2 mg/ml) were isolated using RIPA lysis buffer and then incubated with diluted serum (1:5) collected from *T. thermophila*–injected groupers at 4°C for 12 h. The antigen-antibody complex was immunoprecipitated by a mouse anti-grouper IgM monoclonal antibody (mAb) affinity column (17) and washed with PBS. The complex was analyzed using liquid chromatography–tandem mass spectrometry and mapped to the ciliate proteome database (see schematic diagram in **Figure 1**).

### Protein Structure and Phylogenetic Analysis of *T. thermophila* Tubulin

The amino acid identity of *T. thermophila* tubulin (Tt-tubulin) with ciliate or grouper tubulin was analyzed by the BLAST program. The amino acid sequence of Tt-tubulin was aligned with other animals using the CLUSTALW server (http://www.ebi.ac.uk/clustalw). The three-dimensional (3D) model of *T. thermophila* and *C. irritans* tubulin was built using the SWISS-MODEL server (https://swissmodel.expasy.org/interactive). Phylogenic analysis between ciliates and teleosts was performed using the MEGA 5.04 program. All accession numbers of the sequences used in this part are listed in **Supplementary Table 1**.

### Expression of Recombinant Tt-Tubulin and Antibody Development

A codon optimized Tt-tubulin sequence (XP_001023006) for *Escherichia coli* expression was synthesized by Generay Biotech Co., Ltd. (Shanghai) and then cloned into the pET32a-ΔTRX expression vector (prepared by this lab) to generate pET32a-ΔTRX-Tt-tubulin. The pET32a-ΔTRX-Tt-tubulin plasmid was isolated and transformed into *E. coli* BL21(DE3) cells. The positive bacterial cells were induced with 1 mM isopropyl b-d-1-thiogalactopyranoside (IPTG). Recombinant Tt-tubulin protein (rTt-tubulin) was purified using a nickel nitrilotriacetic acid column (Ni-NTA; Qiagen, Germany).

The purified rTt-tubulin was emulsified with FCA, and 1 mg of protein was injected into New Zealand white rabbits (weighing approximately 1.3 kg). The animals were then boosted with 0.5 mg of rTt-tubulin in FIA on two separate occasions. Serum was then prepared, and the polyclonal antibody (pAb) titers were determined by enzyme-linked immunosorbent assays (ELISAs) using rTt-tubulin as the antigen. Rabbit immunoglobulin G (IgG) was purified from rabbit antiserum using protein A agarose (Beyotime, Haimen, Jiangsu, China) according to the manufacturer’s instructions.

### Sampling of Serum, Mucus, and Tissue Explant Culture

To study the immune response of rTt-tubulin vaccination, all samples for the subsequent analysis were collected at day 3 after challenge. For serum collection, blood was obtained using a 1-ml sterilized syringe *via* the caudal vein from MS-222–anesthetized groupers, and the blood was stored at room temperature for 2 h. Sera were collected by centrifugation at 800 × g for 10 min at 4°C. For skin mucus collection, skin mucus was gently scraped from anesthetized groupers using a clean slide in a 1.5-ml Eppendorf tube. After vigorously vortexing, the supernatant (skin mucus) was harvested by centrifugation at 10,000 × g for 10 min at 4°C and stored at −80°C. Grouper tissue explant culture was performed as previously described (5) with some modifications. Groupers were sacrificed by an overdose of MS-222, and skin, gill, spleen, and head kidney tissues were collected and washed with cold PBS to exclude the blood content. Each tissue was cut into approximately 30-mg portions, placed in a 24-well plate with AIM-V serum-free medium (supplemented with penicillin of 100 U/ml and streptomycin of 100 mg/ml) and then cultured at 28°C for 8 h. The culture medium was collected and centrifuged at 10,000 × g for 10 min at 4°C. A final dose of 1 mM phenylmethylsulfonyl fluoride was added into all collected samples, including serum, mucus, and cultured medium, and the samples were then stored at −80°C.

### ELISA

The purified rTt-tubulin was diluted with coating buffer (pH 9.6) at a dose of 1 μg/ml and then used to coat the wells in a 96-well plate followed by incubation at 37°C for 4 h. Each well was washed with PBST (PBS containing 0.1% Tween) and incubated with blocking buffer (PBST containing 3% BSA) at 4°C for 12 h. After washing with PBST, serum or purified IgG was diluted with dilution buffer (PBST containing 1% BSA) and added into each well at 37°C for 1 h. For the rabbit IgG antibody titer analysis, the wells were detected with horseradish peroxidase (HRP)-conjugated anti-rabbit IgG [1 μg/ml, Cell Signaling Technology (CST)]. For the grouper IgM antibody titer analysis, samples of diluted serum (doubling dilution), mucus (1:5), and tissue culture medium (1:1) were added into each well and incubated at 28°C for 1 h. The wells were then detected with anti-IgM mAb (1 μg/ml) followed by incubation with a HRP-conjugated anti-mouse antibody (1 μg/ml, CST). All wells were incubated with substrate solution, and the optical density at 450 nm (OD450) value was measured using a microplate reader. The last well, in which the value was greater than twice the blank value, was considered as the endpoint titer.

### Parasite-Binding Antibody Detection

The parasite-binding antibody was measured as previously described (8). Briefly, theronts were collected and divided into 10,000 theronts per sample. Theronts were then blocked with incubation buffer (PBS containing 1% BSA) at 28°C for 1 h followed by incubation with serum (1:100 dilution) at 4°C for 12 h with agitation. Theronts were then washed with PBS, lysed with RIPA buffer, and boiled in SDS sample buffer for the subsequent Western blot detection with anti-IgM mAb.

### SDS-PAGE and Western Blot Analysis

Sodium Dodecyl Sulfate PolyAcrylamide Gel Electrophoresis (SDS-PAGE) and Western blot analysis were conducted as previously described (18). Briefly, equal amounts of the protein samples were electrophoresed using a 10% SDS-PAGE gel and transferred to a polyvinylidene fluoride (PVDF) membrane. The PVDF membranes were blocked with 5% skim milk for 1 h at 37°C. For parasite-binding antibody detection, the membranes were incubated with anti-grouper IgM mAb (1 μg/ml) followed by detection with HRP-conjugated anti-mouse IgG (1 μg/ml, CST). For detection of cross-reactive antigens, the membranes were firstly incubated with grouper anti-serum (1:100 dilution) before incubation with anti-grouper IgM mAb. For tubulin detection, the membranes were incubated with anti–rTt-tubulin pAb (1 μg/ml) followed by detection with HRP-conjugated anti-rabbit IgG (1 μg/ml, CST). The resulting bands were visualized using the SuperSignal West Pico Chemiluminescent Substrate.

### Immunofluorescence Staining

*C. irritans* theronts or *T. thermophila* were added dropwise to a slide and fixed with 4% paraformaldehyde (PFA) at 28°C for 15 min. The slides were washed and blocked with 10% goat serum at 37°C for 1 h. For detection of cross-reactive antigens, the slides were firstly incubated with grouper anti-serum (1:100 dilution) followed by incubation with anti-grouper IgM mAb and Alexa 488–conjugated anti-mouse IgG (1 μg/ml, CST). For tubulin detection, the slides were incubated with anti–rTt-tubulin pAb (1 μg/ml) followed by detection with Alexa 488–conjugated anti-rabbit IgG (1 μg/ml, CST). All slides were stained with 1 μg/ml 4′,6-diamidino-2-phenylindole (DAPI, Invitrogen) and mounted in anti-fluorescence quenching agent (Beyotime). All slides were photographed using a NIH-Elements System (Nikon).

### Flow Cytometry

*C. irritans* theronts were collected and fixed with 4% PFA at 28°C for 30 min. Ciliates cells were blocked with 5% goat serum at 28°C for 30 min and stained with anti–Tt-tubulin pAb (rabbit, 1 μg/ml) or normal rabbit IgG (as control) at 28°C for 1 h. After washing with PBS, the Alexa 488–conjugated anti-rabbit IgG secondary antibody (1 μg/ml, CST) was added followed by incubation at 4°C for 1 h. The stained cells were counted by flow cytometry using a CytoFLEX (Beckman) and analyzed by FlowJo software (Tree Star).

### Statistical Analysis

The differences between groups were analyzed by an unpaired Student’s *t*-test, and multiple comparisons were analyzed by the Dunn’s test (Prism version 8.0; GraphPad). *p* < 0.05 was considered statistically significant.

## Results

### Grouper Immunized With *C. irritans* or *T. thermophila* Are Resistant to *C. irritans* Infection

**Figure 2:**
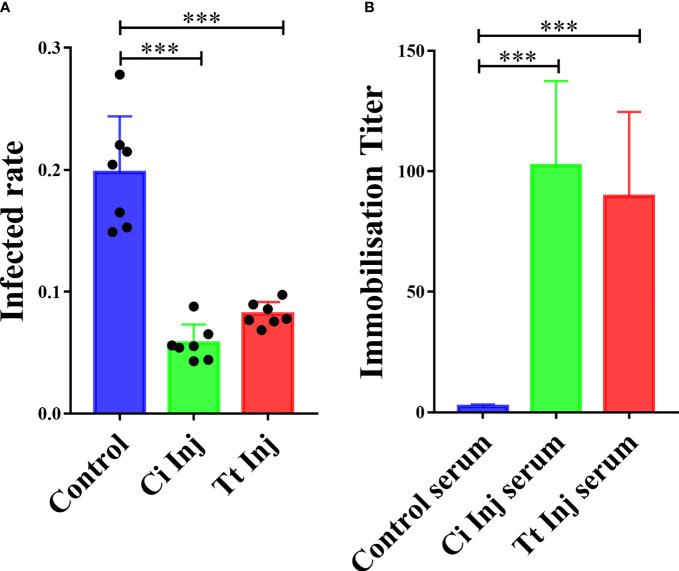
Grouper immunized with *C. irritans* or *T. thermophila* are resistant to *C. irritans* infection. **(A)** *C. irritans* infection rate of *C. irritans–* or *T. thermophila*–immunized groupers (n = 7). Significant differences are represented as *** (p < 0.001). **(B)** Increased immobilization titers were found in serum from *C. irritans–* or *T. thermophila*–immunized groupers (n = 5). Significant differences are represented as *** (p < 0.001).

Previous research has shown that grouper injected with *C. irritans* elicit protective immunity against this parasite’s infection (5). In the present study, we aimed to study whether another ciliate, *T. thermophila*, serves as a heterogenous vaccine and provides immune protection for grouper. After primary and secondary inoculations with *C. irritans* theronts or *T. thermophila* cells, the infection rate was significantly reduced in groupers injected with *C. irritans* theronts (CI group, 5.5%) or *T. thermophila* (TT group, 8.9%) compared to the control group (15.3%) after the challenge **(** **Figure 2A** **)**, which confirmed that vaccination with *T. thermophila* elicited immune protection against the *C. irritans* challenge. Furthermore, an *in vitro* immobilization assay showed that higher titers were detected in the CI and TT group sera, which indicated that both the CI and TT groups generated a specific antibody in their sera **(** **Figure 2B** **)**.

### Identification of Cross-Reactive Antigens Between *C. irritans* and *T. thermophila*

**Figure 3:**
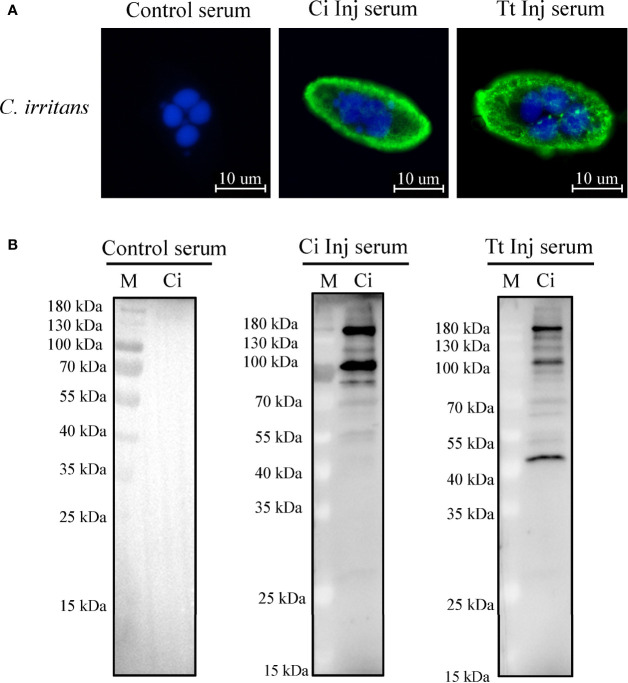
Identification of cross-reactive antigens between *C. irritans* and *T. thermophila*. **(A)** Immunofluorescence staining of *C. irritans* using serum from *C. irritans–* or *T. thermophila–*immunized grouper. **(B)** Western blot analysis of total protein from *C. irritans* and *T. thermophila* using serum from immunized grouper.

To confirm whether any potential cross-reactive antigens exist between *C. irritans* and *T. thermophila*, immunofluorescence staining and Western blot analysis using sera from the CI and TT groups as the primary antibodies were performed. Immunofluorescence staining showed that *C. irritans* was stained with CI or TT group serum but not the control serum **(** **Figure 3A** **)**. Western blot analysis using *C. irritans* theronts as antigens showed that various bands were detected using the CI or TT group serum, and no signal was detected with the control serum **(** **Figure 3B** **)**. Further, to identify the cross-reactive antigens, total protein of *C. irritans* theronts was isolated, incubated with TT group serum, and then immunoprecipitated by an anti-IgM affinity column. After mass spectrometry analysis, some potential cross-reactive antigens were identified **(** **Supplementary Table 1** **)**. The mapping profiles of the top three identified sequences of potential cross-reactive antigens are listed in **Supplementary Figure 1**. Of note, the tubulin molecule had the top score among these potential cross-reactive antigens, which agreed with a previous immune proteomic study (19). In addition, given that tubulin protein is a crucial composition for cilia assembly (20), it may be an effective cross-reactive antigen in ciliates. Thus, the tubulin protein was selected for additional study.

### Characterization of Ciliate Tubulin Protein

**Figure 4:**
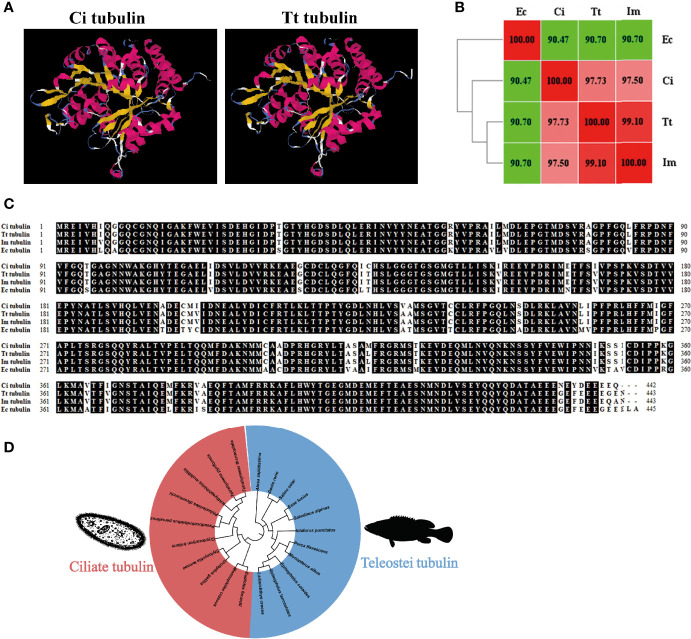
Characterization of ciliate tubulin protein. **(A)** The 3D homology model of *C. irritans* tubulin was performed using the *T. thermophila* tubulin model as a template. **(B)** Heatmap of the amino acid identity between grouper and ciliates. **(C)** Sequence alignment of tubulin between grouper and ciliates. **(D)** Phylogenetic tree between fish and ciliate tubulin. A phylogenetic tree illustrating the relationship between fish and ciliate tubulin using the neighbor-joining method within the MEGA program. Ec, *E. coioides*; Im, *I multifiliis*; Tt, *T. thermophila*; Ci, *C. irritans*.

The predicted 3D model showed that *C. irritans* and *T. thermophila* tubulin share similar structure **(** **Figure 4A** **)**. Homology and alignment analyses showed that *T. thermophila* tubulin shares higher amino acid identity with parasitic ciliates (97%–99%) than grouper (90.7%) **(** **Figures 4B, C** **)**. Furthermore, the phylogenetic tree indicated the formation of two major clades, representing tubulin of ciliates and teleosts **(** **Figure 4D** **)**. Taken together, we hypothesized that ciliate tubulin may serve as a heterologous antigen in fish against parasitic ciliates and that it is less likely to induce an autoimmune response in fish.

### Expression and Antibody Preparation of *T. thermophila* Tubulin

**Figure 5:**
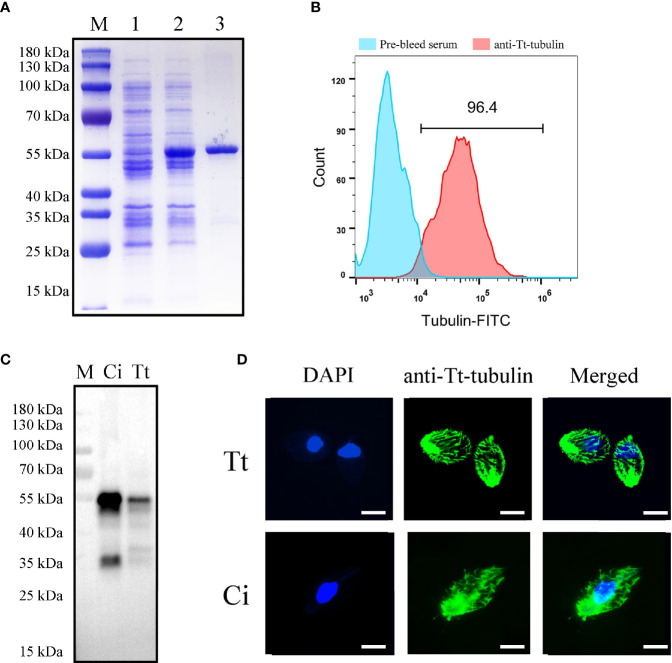
Expression and antibody preparation of *T. thermophila* tubulin. **(A)** SDS-PAGE analysis of purified recombinant *T. thermophila* tubulin in *E. coli*. M, protein marker; Lane 1, non-induced BL21 cells transformed with pET32a-ΔTRX-Tt-tubulin; Lane 2, induced BL21 cells transformed with pET32a-ΔTRX-Tt-tubulin for 4 h; and Lane 3, purified recombinant *T. thermophila* tubulin (approximately 55 kDa) with Ni-NTA. **(B)** Flow cytometry analysis of *C. irritans* using the anti–Tt-tubulin antibody. **(C)** Western blot analysis of total protein from *C. irritans* and *T. thermophila* using the anti–Tt-tubulin antibody. **(D)** Immunofluorescence staining of *C. irritans* and *T. thermophila* using the anti–Tt-tubulin antibody. Scale bar = 10 μm.

As ciliates have a codon usage bias, a codon optimized sequence of *T. thermophila* tubulin adapted to *E. coil* expression was synthesized. After induction with IPTG, an additional band was found after *E. coil* lysis **(** **Figure 5A** **)**. The protein was subsequently purified by a Ni affinity column, and an evident band migrating at approximately 55 kDa was detected by SDS-PAGE followed by Coomassie blue staining **(** **Figure 5A** **)**. High-titer antiserum was prepared by injecting a rabbit with the purified rTt-tubulin. Anti–rTt-tubulin IgG was purified by Protein G affinity column. Flow cytometry analysis showed that more than 96.4% of *C. irritans* theronts were stained with the anti–rTt-tubulin antibody **(** **Figure 5B** **)**. The specificity of the resulting antibody was confirmed by Western blot analysis with two bands of approximately 55 and 35 kDa when the total protein from *C. irritans* theronts or *T. thermophila* cells was screened **(** **Figure 5C** **)**. Immunofluorescence staining of ciliate cells showed that tubulin was diffusely localized in cilia from both *C. irritans* and *T. thermophila* cells **(** **Figure 5D** **)**.

### Tt-Tubulin Provides Immune Protection for Grouper Against *C. irritans* Infection

**Figure 6:**
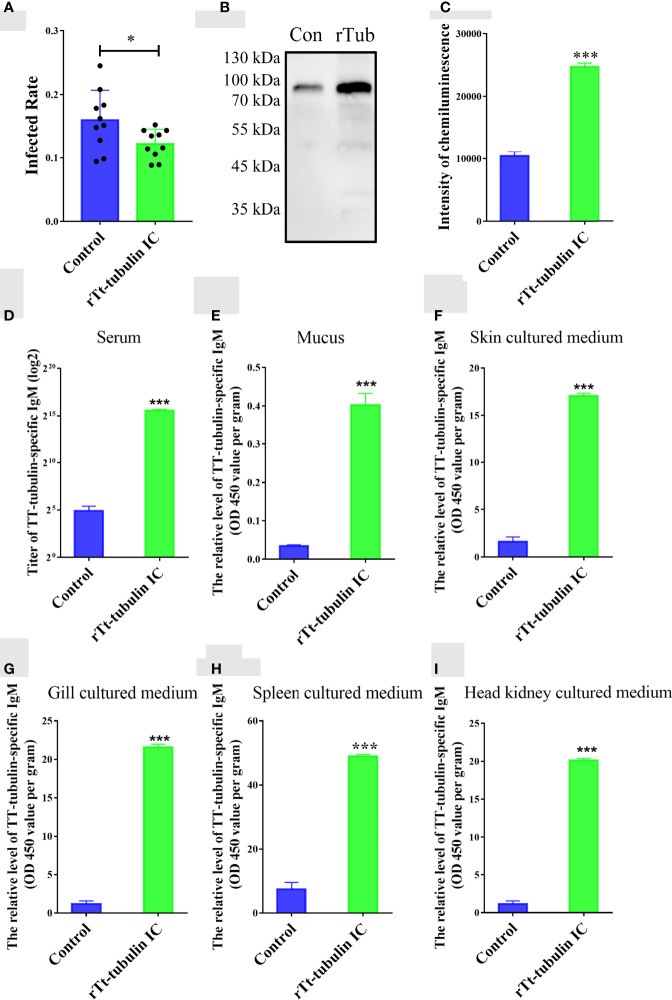
Tt-tubulin provides immune protection for grouper against *C. irritans* infection. **(A)** *C. irritans* infection rate of rTt-tubulin–immunized groupers (n = 10). **(B)** Western blot analysis and **(C)** densitometric analysis of parasite-binding IgM in sera from control and rTt-tubulin–immunized groupers at 72 h after *C. irritans* infection (n = 3). (D–I) ELISA detection of the anti–rTt-tubulin IgM antibody in serum **(D)**, mucus **(E)**, skin culture medium **(F)**, gill culture medium **(G)**, spleen culture medium **(H)**, and head kidney culture medium **(I)** (n = 5). Significant differences are represented as * (p < 0.05) and *** (p < 0.001).

To verify whether *T. thermophila* tubulin contributes to the immune protection against *C. irritans* infection, orange-spotted groupers were injected with rTt-tubulin and challenged with *C. irritans* for 4 weeks after the secondary immunization. The infection rate was significantly reduced in groupers injected with rTt-tubulin (11.4%) compared to the control group (14.8%) after the challenge **(** **Figure 6A** **)**. To study parasite-specific IgM antibody responses in groupers, serum, mucus, and tissue culture medium samples were collected from the control groupers and rTt-tubulin–vaccinated groupers at day 3 after challenge. The parasite-binding antibody was pulled down by theronts, and a specific IgM antibody was detected by Western blot analysis. As expected, an increment of parasite-specific antibodies was detected in rTt-tubulin–vaccinated grouper serum compared to the control grouper serum **(** **Figures 6B, C** **)**. To further analyze the generation of a specific antibody in vaccinated grouper, ELISAs using rTt-tubulin as the antigen were conducted. Similarly, a higher antibody titer was detected in rTt-tubulin–vaccinated grouper serum compared to control grouper serum **(** **Figure 6D** **)**. As the level of antibody is relatively low in mucus and tissue culture medium, the specific antibody level in these samples is presented as an OD value. As expected, higher anti–rTt-tubulin specific antibody levels were found in the mucus and culture medium samples from the skin, gill, spleen, and head kidney from rTt-tubulin–vaccinated groupers compared to the control groupers **(** **Figures 6E–I** **)**.

## Discussion

Many studies have suggested that vaccination may be an effective way to control *C. irritans* infection, and some vaccines, such as inactivated *C. irritans*, DNA, or recombinant subunit vaccines, have been developed (4, 5, 11, 13, 21). However, large-scale production of these vaccines is costly. Thus, heterogenous vaccines may help solve this problem. It has been confirmed that cross-reactive protection is induced by vaccination with free-living ciliates, which are easy to grow in inexpensive culture media (11–13). In the present study, we found that immunization with *T. thermophila* provided grouper with protective immunity against *C. irritans* infection. The higher immobilization titer detected in immunized grouper serum suggested that an anti-parasite antibody was generated after inoculation and that cross-reactive antigens existed between *C. irritans* and *T. thermophila*. This hypothesis was investigated by screening *C. irritans* using *T. thermophila–*immunized grouper serum.

To identify the antigen in *T. thermophila* that contributes to the cross-reactive immunity, immunoprecipitation and mass spectrometry analyses were performed. We identified a *C. irritans* tubulin protein that was immunoprecipitated by the *T. thermophila*–immunized grouper IgM antibody. By immune proteomic screening, tubulin has been determined as one of the candidates for vaccine development against *C. irritans* (19). In addition, recombinant β-tubulin has been suggested as a target antigen to control scuticociliatosis, which is caused by parasitic ciliates (22). I-antigens are surface membrane proteins present on ciliates, and they are considered as the primary vaccine candidates against parasitic ciliates (23–25). However, as the protective immunity induced by I-antigens is serotype specific (26, 27), vaccination based on I-antigens may not cover different *C. irritans* serotypes existing in nature. Tubulin is a well-conserved protein in ciliates, and vaccines using this molecule may solve the problem raised by different serotypes (22). As expected, grouper immunized with rTt-tubulin showed induced protective immunity, but the protective rate was lower than that of *T. thermophila*–immunized grouper, which may due to other antigens existing in *T. thermophila* that may provide immunity stimulation.

Immunofluorescence staining showed that the surface cilia on *C. irritans* were stained by the antibody raised against rTt-tubulin. The large numbers of cilia present on the their surface is an identical structure characterized by ciliates (20), suggesting that a specific antibody targeting cilia may be an alternative method for eliminating parasitic ciliate infection. As tubulin is crucial for cilia assembly, the protection induced by rTt-tubulin was most likely due to the specific anti-tubulin antibody development in grouper. As expected, an increased level of parasite-binding IgMs was detected in rTt-tubulin–immunized grouper serum. Moreover, specific antibodies were also found in mucus and tissue culture medium from rTt-tubulin–immunized grouper. These data confirmed that the rTt-tubulin is a protective cross-reactive antigen, which may be a promising candidate for vaccine development.

In conclusion, vaccination with *T. thermophila* provided groupers immune protection against the *C. irritans* challenge. In addition, tubulin is one of the protective cross-reactive antigens contributing to this process, which stimulates the production of a specific antibody against *C. irritans*. Thus, using *T. thermophila* as a heterologous antigen is a potential method for vaccine development against *C. irritans*.

## Data Availability Statement

The raw data supporting the conclusions of this article will be made available by the authors, without undue reservation.

## Ethics Statement

The animal study was reviewed and approved by the Animal Administration and Ethics Committee of College of Marine Sciences, South China Agricultural University.

## Author Contributions

Conceptualization: YL, XD, and ZM; Investigation: ZM, XL, HW, YH, WG, and YD; Methodology: ZM and HW; Project administration: XD and YL; Supervision: XD; Visualization: ZM; Writing—original draft: ZM and YL; Writing—review and editing: XD, YL, and ZM. All authors contributed to the article and approved the submitted version.

## Funding

This work was supported by the National Natural Science Foundation of China (42006100, 41976081, and 41876162), the Guangdong Basic and Applied Basic Research Foundation (2019A1515110424), the China Postdoctoral Science Foundation (2019M662938), the Guangdong Provincial Special Fund for Modern Agriculture Industry Technology Innovation Teams (2019KJ141), and the China Modern Agricultural Industry Technology System (The Control of Parasites Infection on Marine Fish, CARS-47-18).

## Conflict of Interest

The authors declare that the research was conducted in the absence of any commercial or financial relationships that could be construed as a potential conflict of interest.

## Publisher’s Note

All claims expressed in this article are solely those of the authors and do not necessarily represent those of their affiliated organizations, or those of the publisher, the editors and the reviewers. Any product that may be evaluated in this article, or claim that may be made by its manufacturer, is not guaranteed or endorsed by the publisher.
