# Supplementary figures and images for: Protection of Grouper Against Cryptocaryon irritans by Immunization With Tetrahymena thermophila and Protective Cross-Reactive Antigen Identification
Source: Front Immunol. 2022 Jul 7;13:891643. doi: 10.3389/fimmu.2022.891643 (PMC9300909; doi:10.3389/fimmu.2022.891643)

Top 3 identified sequences mapping profile of potential cross antigens.


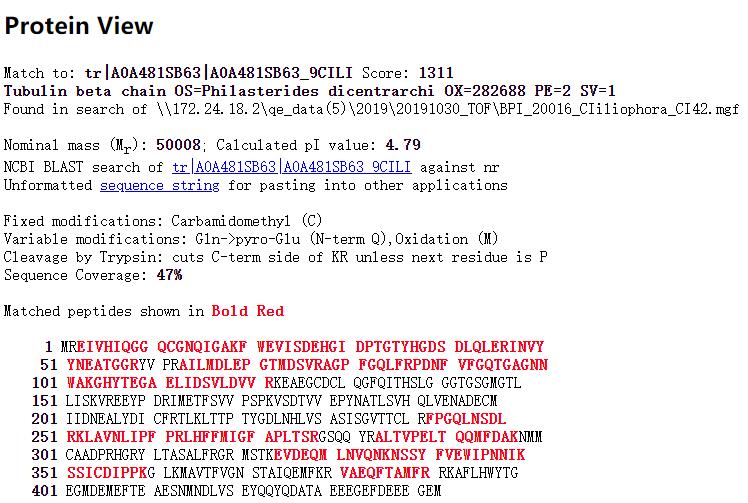

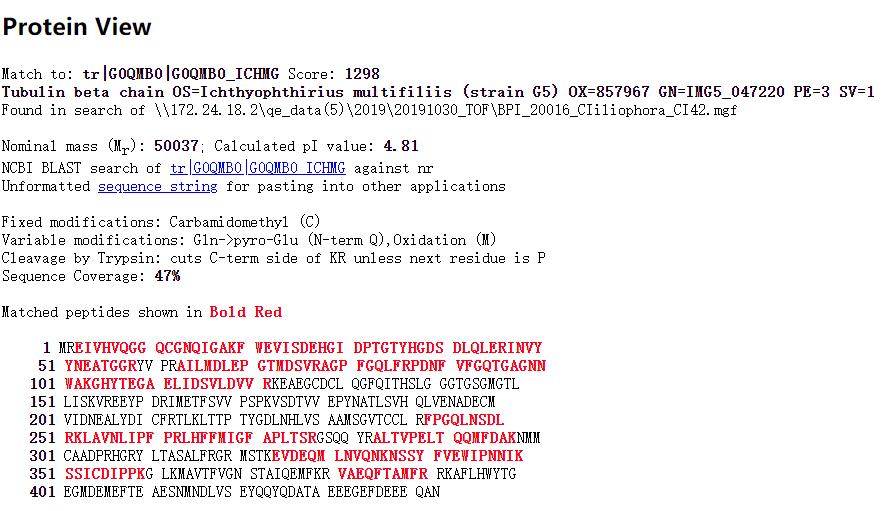

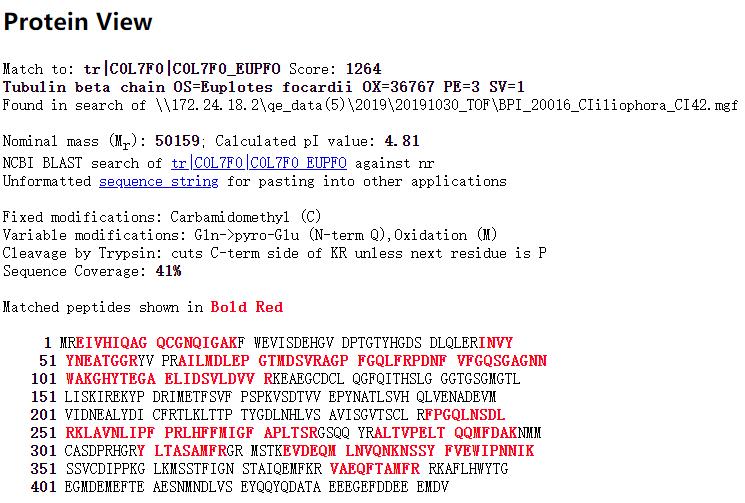

Supplement: Supplementary file 1 [file DataSheet_1.docx]
